# Genome-wide gene-based analysis of rheumatoid arthritis-associated interaction with *PTPN22 *and *HLA-DRB1*

**DOI:** 10.1186/1753-6561-3-s7-s132

**Published:** 2009-12-15

**Authors:** Bo Qiao, Chien Hsun Huang, Lei Cong, Jun Xie, Shaw-Hwa Lo, Tian Zheng

**Affiliations:** 1 Department of Statistics, Columbia University, 1255 Amsterdam Avenue, 10th Floor, MC4690, New York, New York 10027, USA; 2 Department of Statistics, Purdue University, 250 North University Street, West Lafayette, Indiana 47907, USA

## Abstract

The genes *PTPN22 *and *HLA-DRB1 *have been found by a number of studies to confer an increased risk for rheumatoid arthritis (RA), which indicates that both genes play an important role in RA etiology. It is believed that they not only have strong association with RA individually, but also interact with other related genes that have not been found to have predisposing RA mutations. In this paper, we conduct genome-wide searches for RA-associated gene-gene interactions that involve *PTPN22 *or *HLA-DRB1 *using the Genetic Analysis Workshop 16 Problem 1 data from the North American Rheumatoid Arthritis Consortium. *MGC13017*, *HSPCAL3*, *MIA*, *PTPNS1L*, and *IGLVI-70*, which showed association with RA in previous studies, have been confirmed in our analysis.

## Background

Rheumatoid arthritis (RA), MIM 180300, is a symmetric, chronic polyarticular arthritis that affects 0.5-1% of the population. It primarily causes progressive joint destruction that leads to restriction of daily activities and deterioration of quality of life. Although the pathogenesis of RA has not been fully explained, it is thought to be a complex disease caused by a combination of multiple genetic and environmental factors. Previous studies have indicated a few genetic regions that might be in association with RA, most notably *HLA-DRB1 *and *PTPN22*. The *HLA *region on 6p21 is well known for showing the strongest association with RA. The evidence so far has particularly emphasized to the *HLA-DRB1 *gene [1]. The *PTPN22 *gene (1p13.3-p13.1) has also repeatedly shown association to RA, in a modest way [2]. Despite these findings, progress in identifying new genes associated with the susceptibility to RA has been limited. An explanation is that individual contribution of each gene might be small and the traditional methods only test each marker individually, considering no interaction between markers. It is believed that important information is ignored in such an analysis. In this paper, new gene-based methods are used to study gene-gene interactions that involve *HLA-DRB1 *or *PTPN22 *on a whole-genome scale. Association scores between pair-wise single-nucleotide polymorphism (SNP)-SNP interactions and the RA affection status are aggregated into measures of gene-gene interaction effects, using the Genetic Analysis Workshop 16 Problem 1 whole-genome data.

Data collected in a whole-genome association study usually consist of dense SNPs, with multiple SNPs covering a gene. SNP-based analysis is pruned for noise and local dependence, at the same time increasing computational burden. Lo et al. [3] carried out a gene-based analysis on breast cancer candidate genes and demonstrated that using genes as analysis units leads to more stable results and higher interpretability. In this paper, we use a similar gene-based analysis to study interactions between *HLA-DRB1 *or *PTPN22 *and each of the 23,580 genes in the genome for association with RA susceptibility.

## Methods

### Data

The data from Genetic Analysis Workshop 16 Problem 1 consist of 868 cases and 1194 controls from the North Rheumatoid Arthritis Consortium (NARAC), with genotypes on 545,080 SNPs. The percentage of missing data was not high, so we were able to impute the missing values using the computer program fastPhase [4].

Using the whole-genome map of breast cancer data from Cancer Genetic Markets Susceptibility [5], RA SNPs were mapped to genes. SNPs that did not belong to any genes were ignored and different genes with overlapping SNPs were considered separately. For example, if all the SNPs of gene A had been included in gene B, we only considered the "bigger" gene B. After the mapping, we identified 23,580 genes on the 22 chromosomes, including *HLA-DRB1 *(5 SNPs) and *PTPN22 *(12 SNPs). For each of these 23,580 genes, we then studied the interaction with *HLA-DRB1 *or *PTPN22 *in terms of association with RA affection status.

### Association measure of gene-gene interaction

We first introduce the genotype-trait distortion statistic [6] that measures the joint association of multiple SNPs with a disease outcome. Considering *k *SNPs, each with three possible genotypes, there are 3*k *possible genotypes. Genotype-trait distortion is defined as follows

where *n*_*D*, *n*_, *n*_*u*, *s *_are counts of cases and controls with genotype *s*, and *n*_*D*_, *n*_*u *_are the total number of cases and controls. When applied to a single SNP, ***v ***measures the marginal effect of a SNP. If the joint effect of two SNPs exceeds their individual marginal effects, we regard it as an evidence of interaction. As used in Lo et al. [3] to capture interaction signals at SNP level, we define the SNP-level ratio statistic as

where *i*_*d *_is the *d*^th ^SNP of gene *i*, *j*_*e *_is the *e*^th ^SNP of gene *j*, and "∨ "means maximum of the two values. Here  captures the marginal effects of SNPs *i*_*d *_and *j*_*e*_.

To evaluate the effect of each gene pair (*i*, *j*), we define the following gene-level statistics for the marginal effect and interaction signal. Assume gene *i *has *m*_*i *_SNPs and gene *j *has *m*_*j *_SNPs. The first statistic for the marginal effect, called the average maximum marginal *M*, is

The statistic for gene-gene interaction based on the SNP level ratios is defined as

This term is called the "mean interaction ratio" between two genes, or simply, the mean ratio.

### Evaluation of significance

Because the distribution of the mean ratio statistic is unknown, we used permutations to evaluate the significance of the gene-gene interactions. We carried out 1000 permutations and calculated the statistics described above on each *HLA-DRB1*-gene pair and *PTPN22*-gene pair using the original data and the permuted data. Because *HLA-DRB1 *and *PTPN22 *have different numbers of SNPs and potentially different local dependence among the SNPs, we evaluated the gene pairs with *HLA-DRB1 *or *PTPN22 *separately.

Using results from the permuted data, we observe that the magnitude of the gene-gene interaction measures (i.e., mean ratio) depends on the magnitude of the marginal effects under the null hypothesis. Therefore, the evaluation of significance for interaction between a pair of genes should be conditioning on their marginal effect values. We proceeded with a two-dimensional graph of *M*_*ij *_against *R*_*ij *_on the permuted data. This gave us 1000 sets of 23,580 points to create a joint distribution of *M *and *R *under the null hypothesis of no association. We first used the following curve method [3] to construct significance threshold for gene-gene interactions that depends on the values of the marginal effect statistic *M*.

### Curve method

We placed all points (23,580 × 1000 = 23,580,000 points) obtained from the 1000 permutations on the (*M*, *R*) plane. The values of the *M *coordinate were separated into 10,000 bins, each with 2358 points. Each bin was represented by a point with coordinates (M*, R*), where R* was the 99.9% percentile of the *R *scores that fell into this bin and M* was the midpoint of this bin. A smoothing curve, using a spline with 10 degrees of freedom, was fitted to these 10,000 points that represented the 99.9% significance threshold at each *M *level. Gene pairs (*i*, *j*) with (*M*, *R*) coordinates above this curve were selected as significant interactions.

Lo et al. [3] also proposed a rank method for evaluating significance that has lesser distribution assumption than the curve method described above. We used both methods to evaluate the significance of gene-gene interactions.

### Rank method

The rank method also depends on the bins based on the marginal *M *values as the curve method does. Within each bin, we assigned rank values to the 2358 points according to their *R *values (decreasing order: the highest value receives rank 1). For each gene pair, we compared the rank received by the score based on the original data with the ranks received by the permuted values. The *p*-value of this gene pair was then the proportion of the permuted values with rank values lower than the observed data.

## Results

**Figure 1:**
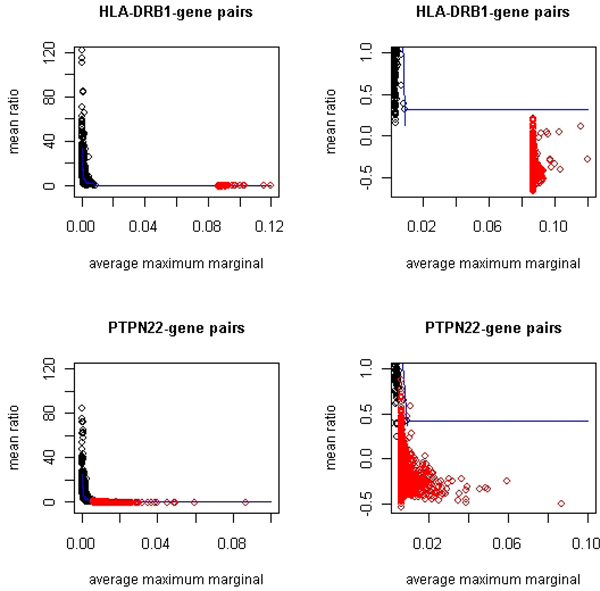
**Observed (*M*, *R*) plane with curve threshold computed from permutations**. Red dots are observed values (*M*, *R*) for gene pairs studied. The black dots are the 99.9% threshold for each bin calculated from 1000 permutations. The dark blue curve is the smoothing spline fitted to the 99.9% bin-thresholds. The right panel displays the same data as the left column with smaller scale to focus on the tail of the scatter.

Figure 1 displays the two-dimensional (*M*, *R*) plane for gene-*HLA-DRB1 *interactions and gene-*PTPN22 *interactions. The observed data are plotted using red dots. The black dots are the threshold calculated within 10,000 bins by the curve method using permuted data as described in the previous section. For interactions with *HLA-DRB1*, the marginal effect is much greater than that is simulated in permutations. This is due to strong marginal signals at *HLA-DRB1 *in the current data. As a result, we had no permuted points that were close to the observed points for calculating reliable selection threshold conditional on the observed *M *values. Therefore, we extended the curve threshold of the last bin to the levels of observed *M *values. This is a conservative extrapolation because the threshold decreases as *M *increases. Based on this conservative threshold, zero gene-pairs were selected to have significant interaction with *HLADRB1*. The rank method, on the other hand, suggested 79 genes to be significant at 0.1% level. However, because the rank values of the observed values were based on the last wide bin that stretched from the top 0.01% of the permutations to the maximum of the observed values, the adjustment to local distribution by using the rank method was not reliable. Due to this concern, in this paper we report only results based on the curve method.

For interaction with *PTPN22*, we also observed strong marginal signals from *PTPN22 *but they are not so dominating as *HLA-DRB1*. The distribution of (*M*, *R*) points from the permutations overlapped slightly with the scatter of the observed data, which allowed us to derive a reliable selection threshold for gene pairs with relatively lower observed *M *values. Using the curve method, 24 genes were selected to have significant interaction with *PTPN22 *at 0.1% level (Table 1). The loci of these genes overlap with important RA susceptibility loci at 5q31 and 16p13. *MGC13017 *(official symbol: *CDKN2AIPNL*) is *CDKN2A *interacting protein N-terminal like according to the NCBI gene database, while *CDKN2A *has been found to encode proteins that inhibit *CDK4 *kinase. Nonomura et al. [7] studied the role of *CDK4 *in the production of inflammatory molecules among subjects with RA and concluded that this protein was associated with the mediation of inflammation. *HSPCAL3 *(official symbol: *HSP90AA2*) is inferred as heat shock protein 90-kDa alpha (cytosolic), class A member 2. Hayem et al. [8] studied stress proteins such as *HSP90 kDa *in the serum of RA patients and found that it may be related to articular prognosis in RA. In the recent study by Vandooren et al. [9], the regulation of *MIA *in RA was observed and studied. *PTPNS1L *is inferred to be protein tyrosine phosphatase, nonreceptor type substrate 1-like and potentially has similar but unknown functionality as some proteins in the same family as *PTPN22*. *IGLVI-70*, at the same locus of *PTPNS1L*, has been thought to be related to the immunoglobulin proteins, which have been found to play important roles in the RA pathology.

**Table 1:** Genes that demonstrates significant RA-associated interaction with *PTPN22 *by the Curve method

Gene	Locus
*LOC115110*	1p36.32
*TAF1A*	1q42
*DKFZP547N043*^a^	1q42.12-q43
*EXOC8*	1q42.2
*HIRIP5*	2p15-p13
*OR7E91P*	2p13.3
*HRB*	2q32.3
*TESSP5*	3q21.31
*SCAP*	3p21.31
*LOC389151*	3q22.3
*TEB4*	5p15.2
*FOXD1*	5q12-q13
***MGC13017* ^b^**	**5q31.1**
*LOC389435*	6q25.2
*LOC441573*	10q23.33
*LOC159770*	11p14.1
***HSPCAL3***	11p14.1
*MGC24665*	**16p13.13**
*NOB1P*	16q22.3
*LOC440382*	16q22.1
***MIA ***	19q13.32
*RAB4B*	19q13.2
*IGLVI-70*	22q11.2
***PTPNS1L***	22q12.2

^a^Genes in the boxes have SNPs in common.

^b^Bold font indicates possible RA susceptibility genes and loci found in the RA literature.

## Conclusion

In this paper, we studied RA-associated interactions between 23,580 genes on the genome and two important RA candidate genes, *HLA-DRB1 *and *PTPN22*. Interaction signals were first calculated at the SNP level and then aggregated into gene-based measurements. Significance was evaluated using permutations. A number of significant gene-*PTPN22 *interactions were found to be associated with the disease status of RA, some of which agreed with previous studies. Through this project, we also found the methods of Lo et al. [3] work better when the marginal signals genes were weak. When the marginal effects of the candidate gene are extremely strong, for example, the odds ratio is greater than 6, the interaction with other genes becomes harder to capture. Further research is needed to address this issue.

## List of abbreviations used

RA: Rheumatoid arthritis; SNP: Single-nucleotide polymorphism.

## Competing interests

The authors declare that they have no competing interests.

## Authors' contributions

S-HL and TZ designed the research. BQ, CHH, and LC carried out the data analysis. BQ, CHH, S-HL, and TZ discussed and interpreted the results. BQ, JX, and TX prepared the manuscript. All authors read and approved the final manuscript.
